# Accumulation of Saponins in Underground Parts of *Panax vietnamensis* at Different Ages Analyzed by HPLC-UV/ELSD

**DOI:** 10.3390/molecules25133086

**Published:** 2020-07-07

**Authors:** Kim Long Vu-Huynh, Huy Truong Nguyen, Thi Hong Van Le, Chi Thanh Ma, Gwang Jin Lee, Sung Won Kwon, Jeong Hill Park, Minh Duc Nguyen

**Affiliations:** 1 Faculty of Pharmacy, Ton Duc Thang University, Ho Chi Minh City 700000, Vietnam; vuhuynhkimlong@tdtu.edu.vn (K.L.V.-H.); nguyentruonghuy@tdtu.edu.vn (H.T.N.); 2 Faculty of Pharmacy, University of Medicine and Pharmacy at Ho Chi Minh City, Ho Chi Minh City 700000, Vietnam; levan@uphcm.edu.vn (T.H.V.L.); mcthanh@ump.edu.vn (C.T.M.); 3 College of Pharmacy, Seoul National University, Seoul 151-742, Korea; ziny2349@snu.ac.kr (G.J.L.); swkwon@snu.ac.kr (S.W.K.)

**Keywords:** *Panax vietnamensis*, vietnamese ginseng, HPLC-UV/ELSD, accumulation of saponins

## Abstract

*Panax vietnamensis* (PV), a wild *Panax* species discovered in Vietnam in 1973, has been increasingly overexploited due to its economic value and therapeutic uses. This resulted in the development of PV cultivation to meet the market demand. There is little information on the accumulation of saponins in PV during cultivation, but this information could serve as an indication of the appropriate harvest time. In this study we developed an HPLC-UV/ELSD method to simultaneously determine the content of 10 characteristic saponins in PV from 2–7 years old, including G-Rb1, G-Rd, G-Rg1, G-Re, N-R1, M-R1, M-R2, V-R2, V-R11, and p-RT4. The result indicated that from 2 to 5 years, the content of saponins in PV rhizome and radix increase 3.02 and 4.2 times, respectively, whereas from 5 to 7 years, no significant changes were observed. Hence, our study suggests that after 5 years of growth could be considered as an appropriate time for PV to be harvested. Among the analyzed saponins, G-Rg1, G-Rb1, G-Rd, and especially M-R2 were the major saponins that contributed to the change of PV’s saponin content through the years. In addition, the developed and validated HPLC method was proven to be reliable and effective for quality control of PV.

## 1. Introduction

**Figure 1:**
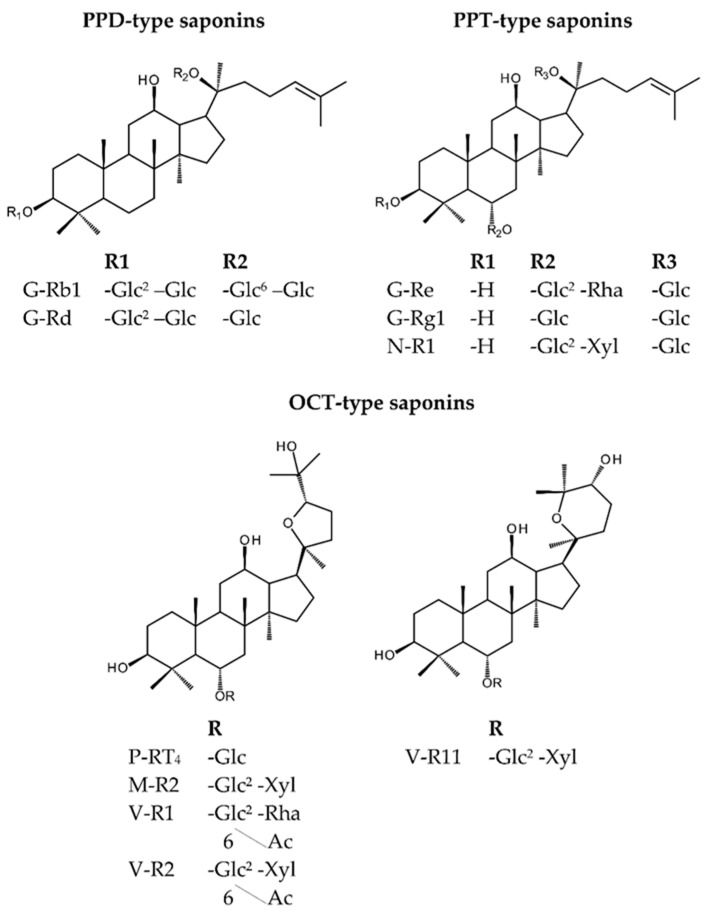
Structures of characteristic saponins in *Panax vietnamensis* (PV).

*Panax vietnamensis* (PV), commonly known as Vietnamese ginseng, was discovered in 1973 in the Ngoc Linh mountainous area, in the middle of Vietnam. The plant has been used by the local Se Dang ethnic minority to enhance physical strength as well as to prevent and cure various diseases. Since its discovery, many investigations on PV in terms of botany, genetics, chemistry, pharmacology, etc., have been carried out. Similar to other well-known *Panax* species such as *Panax ginseng*, *Panax notoginseng*, and *Panax quinquefolius*, the main saponin constituents of PV include dammarane-type saponins with a protopanaxadiol (PPD) skeleton (G-Rb1, G-Rc, G-Rd, etc.) or a protopanaxatriol (PPT) skeleton (G-Rg1, G-Re, N-R1, etc.). However, what makes PV unique from other *Panax* species is its ocotillol-type (OCT) saponins, consisting of M-R1, M-R2, vina-ginsenoside-R1, V-R2, and VR-11 (Figure 1) [1,2,3,4]. Among these characteristic saponins of PV, M-R2 is the major compound with the content of over 5%. Regarding its therapeutic values, PV has been proven to possess a number of biological effects such as anti-oxidative [5], hepato-cytoprotective [6], anti-stress [7], and anti-cancer activities [8,9]. Recently, ocotillol-type saponins from PV were reported to exhibit nephroprotective effects against cisplatin toxicity [10] as well as anti-melanogenic activity [11].

Due to its economic value and therapeutic uses, wild PV has been increasingly overexploited, and thus its natural reserve is seriously in danger of extinction. Consequently, a lot of effort has been focused on the cultivation of PV not only to preserve the natural source but also to meet the growing demand in the market. In ginseng cultivation practice, the content of saponins in the underground parts plays an important role in determining the appropriate time for harvest. For example, Shi, et al. examined the change of saponin contents of *P. ginseng* with growth, in China, from age 1 to 5 years and found a gradual increase of PPD and PPT-type saponin contents with growth year [12]. However, in a study by Li, et al., the variation of ginsenoside contents in *P. ginseng* grown in Korea was higher at the beginning of growth (1–3 years) [13]. As for *P. quinquefolius*, according to Qu, et al. [14], ginsenoside contents changed with age at different rates, G-Re increased gradually with the increase of age. In contrast, the change of G-Rb1 was slight from age 1 to 2 years and significant from age 2 to 5 years. However, up to now, there has been little information on the accumulation of saponins in different parts of PV during cultivation, thus prompting our study.

When it comes to the quantitative assessment of the saponin content in *Panax* species in general, a wide spectrum of analytical methods has been developed so far including HPTLC and HPLC coupled with various detectors such as ultraviolet (UV), diode array detector (DAD), evaporative light scattering detector (ELSD), charged aerosol detector (CAD), and mass spectrometry (MS). Among those, HPLC-DAD or -UV detected at low wavelengths (<205 nm) are frequently used for determination of saponins in *Panax* species [15]. Zhu, et al. also reported the detection and quantitative determination of M-R2, an OCT-type saponin with no double-bond in its structure, at the wavelength of 196 nm with good linearity [16]. In addition, ELSD is another choice for detection of ginsenosides in *P. ginseng* [17] and *P. notoginseng* [18]. This is a mass detection method based on the measurement of scattered light generated by micro-particles transported by the gas flow and directed through a light beam. Li and Fitzloff developed an HPLC-ELSD method for determination of pseudoginsenoside-F11, an OCT-type saponin, with good linearity and precision [19].

In comparison to the analytical method developed for other *Panax* species, analytical methods for quantitative determination of PV have been rarely reported. It is also worth mentioning that OCT-type saponins with no chromophore in their structure bring a challenge for detection. Many attempts were carried out to detect this type of saponin, e.g., using UV at low wavelength (196 nm) [16,20] or a refractive index detector (RID) [21]. RID is a universal detector, independent with the compound structure, which is ideal for detecting poor chromophore compounds. However, there are also many disadvantages of this detector, including low sensitivity, gradient elution incompatibility, susceptibility to contamination, as well as temperature sensitivity. As a result, gradient elution is incompatible with RID, which limits the simultaneous determination of saponins in PV. Different from RID, a UV detector is totally compatible with gradient elution and can detect OCT-types saponins at low wavelength (<200 nm) with an extremely low response. Hence, only M-R2, the most abundant OCT-types saponin, could be detected whereas the others including M-R1, V-R2, V-R11, and p-RT4 were undetectable. Besides, gradient elution coupled with low wavelength detection could cause serious baseline shifts and noise, which interfere the peak integration and quantitative results.

Therefore, in this study, we aimed to develop a reliable and effective method for simultaneous determination of characteristic saponins in PV, especially its unique OCT-type saponins. The developed method was used for analyzing the change of saponin contents in PV roots from age 2–7 years. The results illustrated that at the age of 5, the increase in both weight and saponin contents of PV almost reached their plateau, indicating the appropriate age for harvest. Furthermore, our developed method, which was validated for linearity, precision, accuracy, limit of detection, and limit of quantification, is expected to provide a good analytical method for the quality control of PV and its products.

## 2. Results and Discussions

### 2.1. Development of the HPLC-UV-ELSD Quantitative Method

#### 2.1.1. Optimization of the HPLC Condition

A reasonable elution program of acetonitrile and water was achieved to obtain a good separation profile of 10 saponins. Notably, the separation of G-Rg1 and -Re, which have been a challenge for RP-HPLC analysis of *Panax* species, was solved. A UV detector at the wavelength of 203 nm is usually employed for analysis of PPT- and PPD-type saponins [20]. However, the detection of OCT-saponins, the main constituents of PV at 203 nm, is restricted due to the lack of chromophore in their structures. Therefore, to detect OCT-saponins, UV 196 nm should be used, but the detection at such a low wavelength might be prone to baseline instability and insufficient peak intensity.

To overcome these limitations of UV, ELSD might serve as a better choice for the detection of OCT-type saponins in PV. We compared the performance of ELSD at a nebulizer gas pressure of 3.5 bar and 3.8 bar, and the pressure of 3.8 bar showed better reproducibility of increased peak area by the decrease of the relative standard deviation (% R.S.D.) (Table S1). The design of the detector selects droplets as a function of size. Thus, large droplets, which are more difficult to evaporate and cause an increased noise level, were discarded in the glass nebulization cell. Therefore, the higher nebulizing gas pressure could decrease the size of droplets and allow the eluent to enter the drift tube for detection, which led to an improvement of reproducibility. However, it is worth mentioning that too high a nebulizing gas pressure can permanently damage the drift tube.

**Figure 2:**
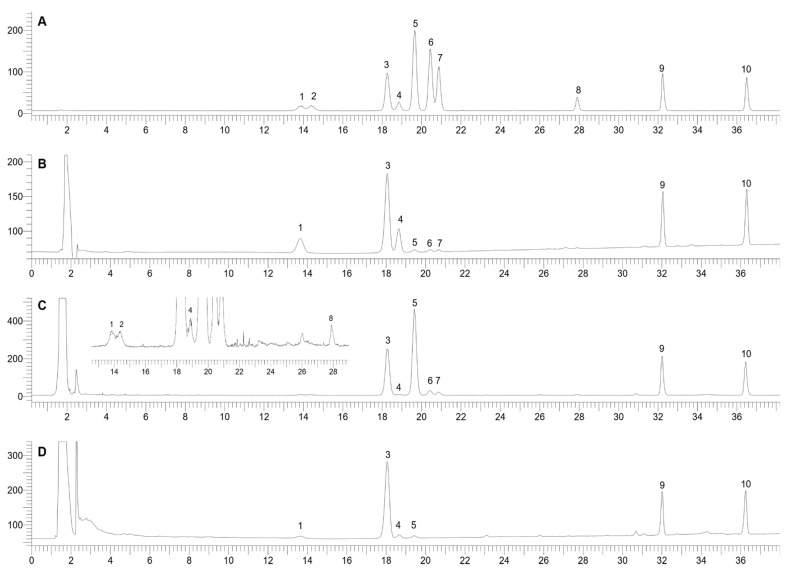
HPLC chromatograms of a standard mixture detected by ELSD (**A**) and UV (**B**); PV extract detected by ELSD (**C**) and UV (**D**). 1, N-R1; 2, M-R1; 3, G-Rg1; 4, G-Re; 5, M-R2; 6, p-RT4; 7, V-R11; 8, V-R2; 9, G-Rb1; 10, G-Rd.

HPLC chromatograms detected by the UV detector and ELSD in the optimized condition are shown in Figure 2. From the chromatograms, it is notable that ELSD could well detect at least ten major saponins in PV, including the characteristic OCT-type saponins of PV, i.e., M-R1, M-R2, V-R2, V-R11, and p-RT4 even in low concentrations. In contrast, UV at 196 nm could only detect six peaks, including those of the major OCT-saponin M-R2, PPT-type saponins (N-R1, G-Rg1, and G-Re) and PPD-type saponins (G-Rb1, and G-Rd). Thus, the UV detector could only detect the major saponin M-R2 with very low peak intensity, but neglected the other OCT-saponins (M-R1, V-R2, V-R11, and p-RT4). This agrees with the advantage of ELSD as a universal detector, which could well detect most of the non-volatile compounds. Hence, the ELSD chromatogram could well reflect the relative correlations of saponin contents in PV extracts. M-R2 is the main constituent of PV extracts, followed by PPT-type G-Rg1, and then the PPD-type saponins G-Rb1 and G-Rd. The other saponins, including N-R1, G-Re, M-R1, V-R2, V-R11, and p-RT4, are minor compounds. In contrast, by not taking into account the quantitative results, the UV chromatogram of the PV extract could suggest erroneously that M-R2 is only a minor component in PV because of its very low-intensity peak.

#### 2.1.2. Optimization of the Extraction Method

Seventy percent MeOH was chosen as the extracting solvent for PV saponins [15,22,23]. To optimize the extraction method, PV powder (100 mg) was suspended with 10 mL of 70% MeOH in capped 15 mL conical tubes and extracted with ultrasound. Variables involved in the procedure such as temperature (30, 60, 80 °C), extraction time (10, 20, 30, 40 min), and extract repetitions (1, 2, 3 times) were optimized. The optimization was based on the peak areas of G-Rg1, G-Rb1, G-Rd, and M-R2, the four most abundant saponins in PV. The content of these four saponins in the extract may closely reflect the extraction efficacy.

**Figure 3:**
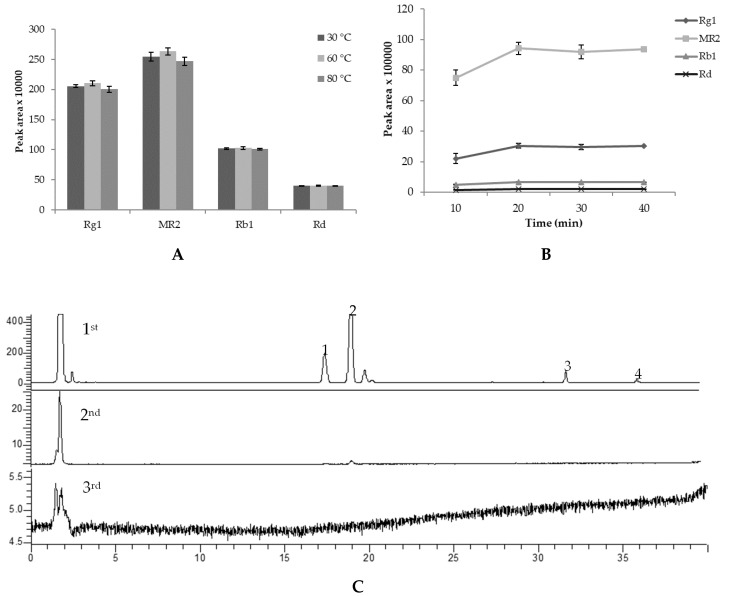
Peak area of main saponins of PV extracted at different extraction temperatures (**A**), duration (**B**), and extraction at 1st, 2nd, and 3rd times (**C**). The results of **A** and **B** expressed at the mean and standard deviation of a triplicated experiment.

Figure 3A showed no significant difference in peak areas of saponins in chromatograms of PV extracts at different extraction temperatures. For the extraction duration, as illustrated in Figure 3B, the content of the main saponins in the extract increased rapidly from 10 to 20 min, but from 20–40 min, no significant change was observed. The first 20 min extraction recovered over 96% of saponins compared with the second and third extractions (Figure 3C). Therefore, single extraction for 20 min at 30 °C was used for the extraction of saponins from PV with the details presented in Figure 3. The optimized extraction method is simple that could significantly save time and solvent. By using this method, many samples could be extracted at the same time, which could increase the analysis efficiency. Specifically, our developed extraction only requires 100 mg of sample powder, which is suitable for high-value samples such as PV.

### 2.2. Comparing UV and ELSD in Quantitative Determination of Saponins in PV

Under the above-mentioned HPLC-ELSD/UV conditions, the performance of both detectors in the detection of saponins in PV were compared. With both ELSD and UV detection, the results showed good linearity (R^2^ ≥ 0.98 in ELSD and R^2^ ≥ 0.9997 in UV detection, Table 1). The UV detector showed a better sensitivity for PPT and PPD saponins with the LOQ in the range of 0.001–0.002 mg/mL and LOD in the range of 0.0002–0.0004 mg/mL while ELSD has lower sensitivity with LOD and LOQ 10–15 times higher than that of the UV detector. However, ELSD showed advances in detection of M-R2 with LOD and LOQ of 0.004 mg/mL and 0.019 mg/mL, respectively, which was two times lower than those of the UV detector. Moreover, ELSD could quantitatively determine other minor OCT-type saponins, including M-R1, V-R2, V-R11, and p-RT4, whereas UV failed to do so.

Table 2 exhibits the reproducibility of the optimized quantitative method using the ELSD and UV detectors presented by % R.S.D. of each compound. With the UV detection, intra- and inter-day variations were less than 2 and 6%, respectively, while those of ELSD were both less than 4%. The developed method had good accuracy with the overall recovery of 89.4–107% in ELSD and of 96–106.3% for UV detection (Table 3). However, some minor saponins such as N-R1, M-R1, and G-Re were detected under the LOQ of ELSD. Therefore, the detection of these saponins is just for qualitative purposes. Regarding the validation data, ELSD showed superior performance and, therefore, was chosen for quantitative determination of saponins in PV for its precision and sensitivity.

### 2.3. Growth Characteristics of the Underground Parts of PV with Age

**Figure 4:**
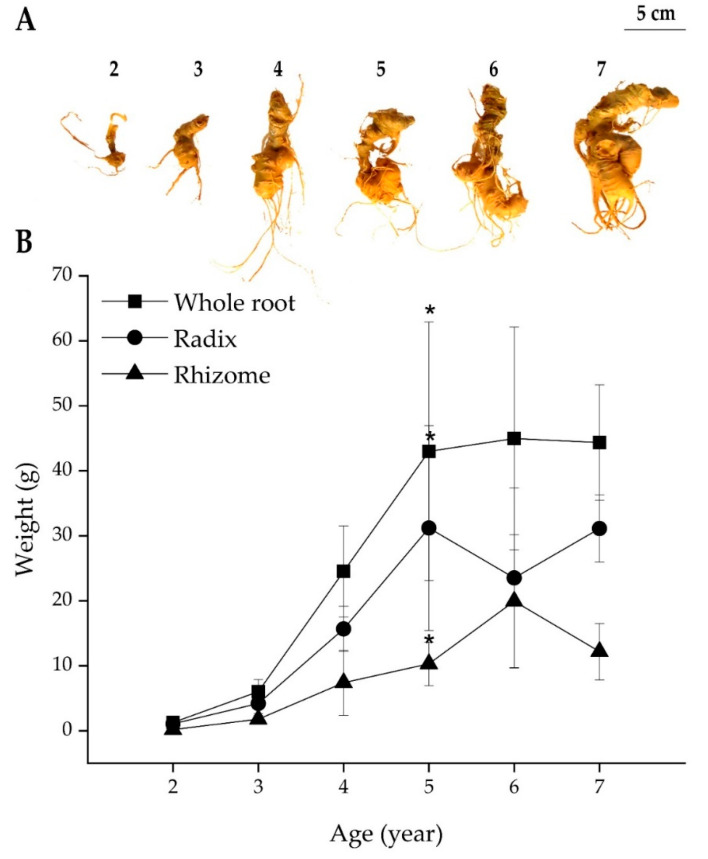
*Panax vietnamensis* underground parts from age 2–7 years (**A**) and the change of whole root, radix, and rhizome fresh weight of PV from 2–7 years (**B**). Results are expressed as mean ± SD (*n* = 5), * *p* < 0.05 compared with that at age 2 years.

As illustrated in Figure 4, the weight of fresh underground parts of PV aged from 2 to 7 years, including whole root, radix, and rhizome, increased rapidly from age 2 to 5 years, and no significant difference was observed after this period. At the maximum growth, the weight of the radix was about 3-fold higher than that of the rhizome and contributed mostly to the increase of whole root weight. Over the first five years of growth, the rhizome weight increased rapidly (55 times, from 0.19 g to 10.29 g) while those of radix and whole root only increased about 29.7 and 33.9 times, respectively. Interestingly, the weight increase of PV root at age five years was similar to that of Korean Ginseng root (35 times), as reported by Li et al. [13]. The results suggest that in terms of weight, similar to Korean Ginseng, a five-year term may be suitable for the commercial cultivation process of PV.

### 2.4. Saponin Contents in the PV Radix at Different Ages

**Figure 5:**
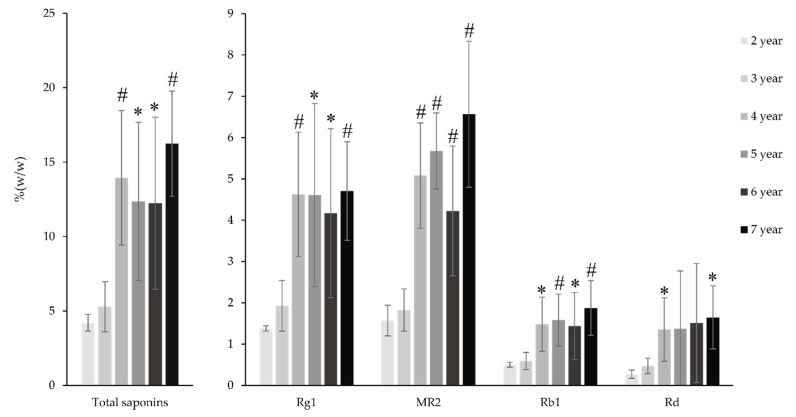
The change of saponin contents in the PV radix from 2–7 years. Results are expressed as mean ± SD (*n* = 5), * *p* < 0.05, # *p* < 0.01 compared with that at the age at two years.

It can be seen from Figure 5 that the content of saponins in the PV radix varied from ages 2–7 years. The total saponin content increased significantly from age 2–4 years (3.3 times) and then changed slowly. G-Rg1 and M-R2 shared a similar accumulation pattern with the total saponin content from age 2–7 years. From age 2–4 years, the increased ratio of M-R2 was 3.24 times, and that of G-Rg1 was 3.23 times. This interesting similarity could be clearly explained by the high proportion of G-Rg1 and M-R2 contents in PV radix (3.67% and 3.70%, respectively). However, G-Rb1 and G-Rd, PPD-type saponins, increased significantly only from age 3–4 years (2.95 and 5.01 times, respectively). From age 4–7 years, the content of these two saponins increased at a slow rate (1.2 times). The content of other minor OCT-type saponins, including V-R11 and V-R2 at the age of 5 were 2.66 and 1.59 times, respectively, as high as those at the age of 2, whereas p-RT4 content remained constant (Table S2). The change of total saponins as well as PPT- and PPD-type saponins of PV were quite similar to those of *P. ginseng*, which increased from age 1 to 4 years [12]. The change of these saponins in *P. quinquefolius* also differed from those of PV, where G-Rb1 increased slightly from 1–2 years and then increased significantly from 2–5 years [14].

### 2.5. Saponins Contents in the PV Rhizome at Different Ages

**Figure 6:**
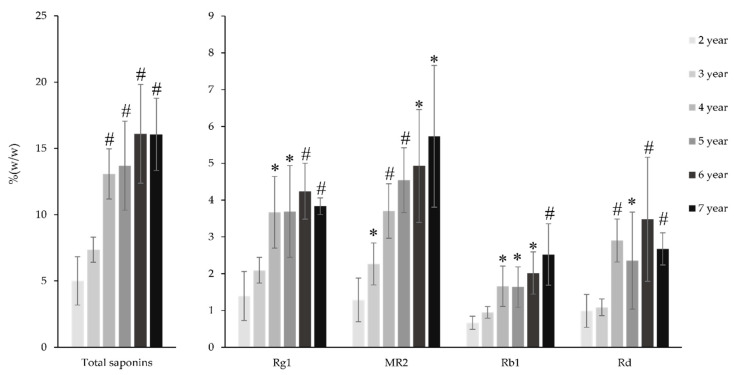
Change of saponin content of the PV rhizome from 2 to 7 years. Results are expressed as mean ± SD (*n* = 5), * *p* < 0.05, ^#^ *p* < 0.01 compared with that at the age of two years.

The increasing pattern of the total saponins content in the PV rhizome was quite similar to those of the radix, which showed an upward trend from 2–4 years and then remained stable (Figure 6). This change was attributable to the change of G-Rg1 and M-R2, the two main saponins in the PV rhizome at six years at 3.67% and 3.70%, respectively. From 2–4 years, the total saponin content of the PV rhizome increased 2.61 times, which is remarkably similar to the increasing rate of G-Rg1 and M-R2 (2.62 and 2.86 times, respectively). From age 4–7 years, the content of M-R2 continuously increased from 3.70% to 5.73% while that of G-Rg1 slightly increased and then decreased. The rate of increase of G-Rb1 saponins is similar to that of M-R2, while G-Rd shared the same accumulation pattern with G-Rg1. At age four years, G-Rb1 and G-Rd contents were 2.67 and 2.92 times higher, respectively, than those at age two years (Table S3). Interestingly, from 6–7 years, the content of G-Rb1 increased from 2.02% to 2.52%, while that of G-Rd decreased rapidly from 3.48% to 2.67%. The contrary changes of these two PPD-type saponins could be explained by the biosynthetic pathway of the ginsenoside. As reviewed by Kim et al., G-Rd could be glycosylated by an enzyme named uridine diphosphate-dependent glycosyltransferase to add a glucose moiety at C20 to yield G-Rb1. Similarly, the decrease of G-Rg1 may also be explained by the glycosylation of this saponin to yield G-Re by adding a rhamnose moiety at the C6 position [24].

## 3. Materials and Methods

### 3.1. Materials and Instrument

PV were cultivated in semi-natural conditions under a forest at Tra Linh Farm, at the height of 1.600 m on Ngoc Linh Mount, Quang Nam Province, Vietnam. The seeds were set in February, and the plants grew naturally in the original forest conditions without any fertilization. The underground parts of fresh 2–7 year old cultivated PV were collected in December, during the winter when PV hibernates. Each age group comprised five different samples. A voucher specimen was deposited in the herbarium of the College of Pharmacy, Seoul National University (SNUP-2014-A-03, Seoul, Korea). The underground parts were cleaned and dried at 50 °C in a drying oven until dry. The dried material was then ground and sieved to give a fine powder with particle size less than 355 µm.

Before conducting this study, reference standards, including G-Rb1, G-Rd, G-Rg1, G-Re, N-R1, M-R1, M-R2, V-R2, V-R11, and p-RT4 were prepared in the laboratory following our previous reports [1,2,3]. HPLC-grade solvents were purchased from J.T. Baker (Deventer, Netherlands).

HPLC analyses were performed on a Perkin Elmer series 200 HPLC system (Perkin Elmer, Inc., Waltham, MA, USA) equipped with vacuum degasser, quaternary gradient pump, auto-sampler, UV-vis detector coupled with Sedex 80LT ELSD (Sedere, Alfortville Cedex, France), connected to a TotalChrom Workstation software. A Gemini C_18_ column (150 mm × 4.6 mm. i.d., 5 µm) and a Gemini C_18_ guard column (Phenomenex, Torrance, CA, USA) were used.

### 3.2. Methods

#### 3.2.1. Sample Preparation

First, 100 mg of 6 years old PV root powder was placed into a 12 mL glass tube containing 10 mL of 70% aqueous methanol. The tightly capped tube was shaken by vortex (Vortex-Genie, Bohemia, NY, USA) for 10 s before being subjected to ultrasonication (Branson 5510, Danbury, CT, USA) for 20 min at 30 °C. During ultrasonication extraction, the tube was also shaken by vortex every 10 min. The extract was then filtered through a 0.45 µm filter prior to HPLC analysis.

#### 3.2.2. HPLC Analysis

The separation was achieved by using a Gemini C_18_ column (150 mm × 4.6 mm. i.d., 5 µm) and a binary gradient elution system consisting of water (A) and acetonitrile (B) with the following gradient program: 0–11 min, 21% B; 11–25 min, 21–32% B; 25–35 min, 32–40% B; 35–40 min, 40–95% B; 40–60, 95% B; 60–61, 95–21% B; 61–71, 21% B. The flow rate was set at 1.0 mL/min, and the injection volume was 20 µL. The column temperature was maintained at 30 °C. The UV detection wavelength was set at 196 nm, and the ELSD was set to the drift tube temperature of 50 °C and the nebulizer gas (N_2_) pressure of 3.8 bar.

Identification and quantification of saponins were carried out by comparing the retention time and the peak area with those of corresponding standards. The saponin amount in whole root was calculated by the following formula: saponin amount in whole root = (saponin contents in rhizome × rhizome weight) + (saponin contents in radix × radix weight).

#### 3.2.3. Calibration Curve

Stock solution containing G-Rb1, Rd, Re, Rg1, N-R1, M-R1, M-R2, V-R2, V-R11, and P-RT4 in aqueous methanol was prepared and diluted to 10 concentrations for the establishment of calibration curves. In the UV detection, the calibration was constructed by plotting the peak areas versus the concentration of each compound, while in the ELSD, the calibration was based on the decimal logarithm of both peak areas and concentrations. The calibration curves constructed for UV and ELSD can be found in Figures S1 and S2, respectively. The LOD and LOQ under the present chromatographic conditions were determined based on the response at signal-to-noise ratios of about 2–3 and 10, respectively.

#### 3.2.4. Validation

The precision of the analyzing method was determined by intra- and inter-day variations. First, 100 mg of PV was extracted and analyzed as described above. The intra-day precision was performed by triplicate extraction and analysis on a single day. The inter-day precision was carried out on three different days. Variations were expressed by the relative standard deviation (% R.S.D.). The recovery test was used to evaluate the accuracy of this quantification method. An accurate amount of each saponin was added to 100 mg of the PV sample and then extracted and analyzed as described. The average recovery was determined by the following formula: recovery (%) = (observed amount − original amount)/spiked amount × 100%, with % R.S.D. = (S.D./mean) × 100%.

#### 3.2.5. Statistical Analysis

The data are presented as the mean ± standard deviation. To compare the saponin content of the two age groups, the Student *t*-test was used with SPSS version 21.0. A *p*-value of <0.05 was considered statistically significant.

## 4. Conclusions

In this study, we successfully developed and validated a reliable and effective HPLC-UV/ELSD method for simultaneous determination of 10 characteristic saponins in the underground parts of *Panax vietnamensis*. The developed method illustrated that the saponin content of PV increased from age 2–7 years, and at the age of 5, the increase in both weight and saponin contents of PV readily reached their plateau, suggesting an appropriate age for harvest. The acquired data also showed the preferable advantage of the ELSD detector over the UV detector in the detection of characteristic saponins of PV, especially its unique OCT saponins, which do not possess chromophore in the structure. As a result, the developed HPLC-ELSD method might serve as a good means for quantitative and qualitative analyses of this ginseng.

## Figures and Tables

**Table 1:** Calibration curve for ten saponins in the ELSD and UV detectors.

**Saponin**	**ELSD**				
	**Equation**	**R^2^**	**Range (mg/mL)**	**LOQ** **(mg/mL)**	**LOD** **(mg/mL)**
N-R1	y = 16,095,158 × x^1.6081^	0.9985	0.013–1.040	0.0220	0.0060
M-R1	y = 18,280,672 × x^1.5972^	0.9804	0.013–1.010	0.0220	0.0060
G-Rg1	y = 14,505,168 × x^1.5687^	0.9983	0.007–0.955	0.0220	0.0070
G-Re	y = 15,540,853 × x^1.6196^	0.9977	0.013–1.075	0.0220	0.0070
M-R2	y = 20,958,157 × x^1.6685^	0.9984	0.019–0.61	0.0190	0.0040
P-RT4	y = 20,123,608 × x^1.6351^	0.9990	0.009–0.56	0.0160	0.0065
V-R11	y = 4,707,057 × x^1.6007^	0.9994	0.055–1.69	0.0550	0.0260
V-R2	y = 22,774,081 × x^1.6510^	0.9994	0.007–0.56	0.0130	0.0035
G-Rb1	y = 30,016,270 × x^1.6916^	0.9991	0.007–0.495	0.0130	0.0038
G-Rd	y = 28,974,603 × x^1.7116^	0.9993	0.007–0.495	0.0120	0.0039
**UV**					
N-R1	y = 8,225,894x − 12,872	1	0.0004–1.0400	0.0020	0.0004
M-R1	ND	ND	ND	ND	ND
G-Rg1	y = 9,069,242x + 12,434	0.9997	0.0019–1.9100	0.0018	0.0004
G-Re	y = 9,069,242x + 12,434	0.9997	0.0019–1.9100	0.0018	0.0004
M-R2	y = 188,174.90x − 166.66	1	0.009–2.4400	0.04	0.010
P-RT4	ND	ND	ND	ND	ND
V-R11	ND	ND	ND	ND	ND
V-R2	ND	ND	ND	ND	ND
G-Rb1	y = 6,675,167x − 221	0.9999	0.00097–0.9900	0.0010	0.0002
G-Rd	y = 7,278,735x + 1457	1	0.00098–1.0000	0.0010	0.0002

ND—not detected.

**Table 2:** Inter- and intra-day precision of the HPLC-ELSD-UV method.

Saponin	ELSD				UV			
	Intra-Day Precision		Inter-Day Precision		Intra-Day Precision		Inter-Day Precision	
	Contents (mg/g)	% R.S.D.	Contents (mg/g)	% R.S.D.	Contents (mg/g)	% R.S.D	Contents (mg/g)	% R.S.D.
N-R1	ND	NA	ND	NA	1.5 ± 0.03	2.01	1.5 ± 0.11	2.24
M-R1	ND	NA	ND	NA	ND	NA	ND	NA
G-Rg1	35.80 ± 0.2	0.75	36.6 ± 1.1	3.03	37.3 ± 0.1	0.27	37.2 ± 0.14	0.378
G-Re	ND	NA	ND	NA	0.89 ± 0.01	1.53	0.9 ± 0.05	5.91
MR2	62.8 ± 0.3	0.51	64.1 ± 1.5	2.43	65.1 ± 1.3	1.99	66.8 ± 1.4	2.20
p-RT4	16.8 ± 0.2	1.4	17.6 ± 0.6	3.78	ND	NA	ND	NA
VR11	6.70 ± 0.2	3.03	7.70 ± 0.18	2.37	ND	NA	ND	NA
VR2	1.50 ± 0.05	3.77	1.70 ± 0.06	3.87	ND	NA	ND	NA
G-Rb1	10.1 ± 0.00	0.09	10.5 ± 0.3	2.96	10.1 ± 0.02	0.2	10.1 ± 0.07	0.75
G-Rd	5.60 ± 0.06	1.14	5.60 ± 0.12	2.26	5.6 ± 0.01	0.27	5.6 ± 0.026	0.46

ND—not detected; NA—not available.

**Table 3:** Accuracy of the HPLC-ELSD-UV method for the determination of saponins in PV.

Saponin	ELSD					UV				
	Original (mg)	Spiked (mg)	Observed (mg)	Recovery (%)	R.S.D. (%)	Original (mg)	Spiked (mg)	Observed (mg)	Recovery (%)	R.S.D. (%)
N-R1	ND	NA				0.15	0.09	0.24	98	6.9
							0.11	0.26	97.8	5.7
M-R1	ND	NA				ND	NA			
G-Rg1	3.66	2.4	5.95	94.8	3.27	3.72	2.4	6.11	99.5	1.66
		3.02	6.69	100.2	1.18		3.02	6.83	100.2	1.14
G-Re	ND	NA				0.09	0.1	0.17	81.6	0.3
							0.12	0.19	84.5	0.4
M-R2	6.41	3.98	10.17	94.3	4.15	ND	NA			
		4.98	11.21	96.5	0.4					
p-RT4	1.71	1.02	2.72	98.5	4.49	ND	NA			
		1.23	2.81	89.4	0.9					
V-R11	0.67	0.52	1.17	111.5	4.06	ND	NA			
		0.62	1.31	102.3	0.42					
V-R2	0.17	0.08	0.25	103.7	5.4	ND	NA			
		0.1	0.28	101	6.36					
G-Rb1	1.01	1.0	1.98	97	2.3	1.01	1.0	2.01	101	1.62
		1.2	2.12	92	7.63		1.2	2.15	96	5.75
G-Rd	0.56	0.52	1.05	95	0.96	0.56	0.52	1.1	106.3	2.86
		0.63	1.13	90	3.07		0.63	1.19	98	6.45

ND—not detected; NA—not available.

## Supplementary Materials

The Supplementary Material are available online.
