# Supplementary material for: Accumulation of Saponins in Underground Parts of Panax vietnamensis at Different Ages Analyzed by HPLC-UV/ELSD
Source: Molecules. 2020 Jul 7;25(13):3086. doi: 10.3390/molecules25133086 (PMC7411744; doi:10.3390/molecules25133086)
Supplement: Supplementary file 1 [file molecules-25-03086-s001.pdf]

Supplementary Material

Accumulation of Saponins in Underground Parts of *Panax Vietnamensis* at Different Ages Analyzed by HPLC-UV/ELSD

Kim Long Vu-Huynh [1,†], Huy Truong Nguyen [1,†], Thi Hong Van Le [2], Chi Thanh Ma [2], Gwang Jin Lee [3], Sung Won Kwon [3], Jeong Hill Park [3,*] and Minh Duc Nguyen [1,*]

[1] Faculty of Pharmacy, Ton Duc Thang University, Ho Chi Minh City 70000, Vietnam; vuhuynhkimlong@tdtu.edu.vn (K.L.V.-H.); nguyentruonghuy@tdtu.edu.vn (H.T-N.); nguyenminhduc@tdtu.edu.vn (M.D-N)

[2] Faculty of Pharmacy, University of Medicine and Pharmacy at Ho Chi Minh City, Ho Chi Minh City 70000, Vietnam; levan@uphcm.edu.vn (T.H.V.L.); mcthanh@ump.edu.vn (C.T.M.)

[3] College of Pharmacy, Seoul National University, Seoul 151–742, Korea; ziny2349@snu.ac.kr (G.J.L), swkwon@snu.ac.kr (S.W.K)

* Correspondence: Correspondence: hillpark@snu.ac.kr (J.H.P.); nguyenminhduc@tdtu.edu.vn (M.D.N.); Tel.: +82-2-880-7857 (J.H.P.); +84-90-898-8820 (M.D-N.)

† Correspondence: These authors contributed equally to this work.

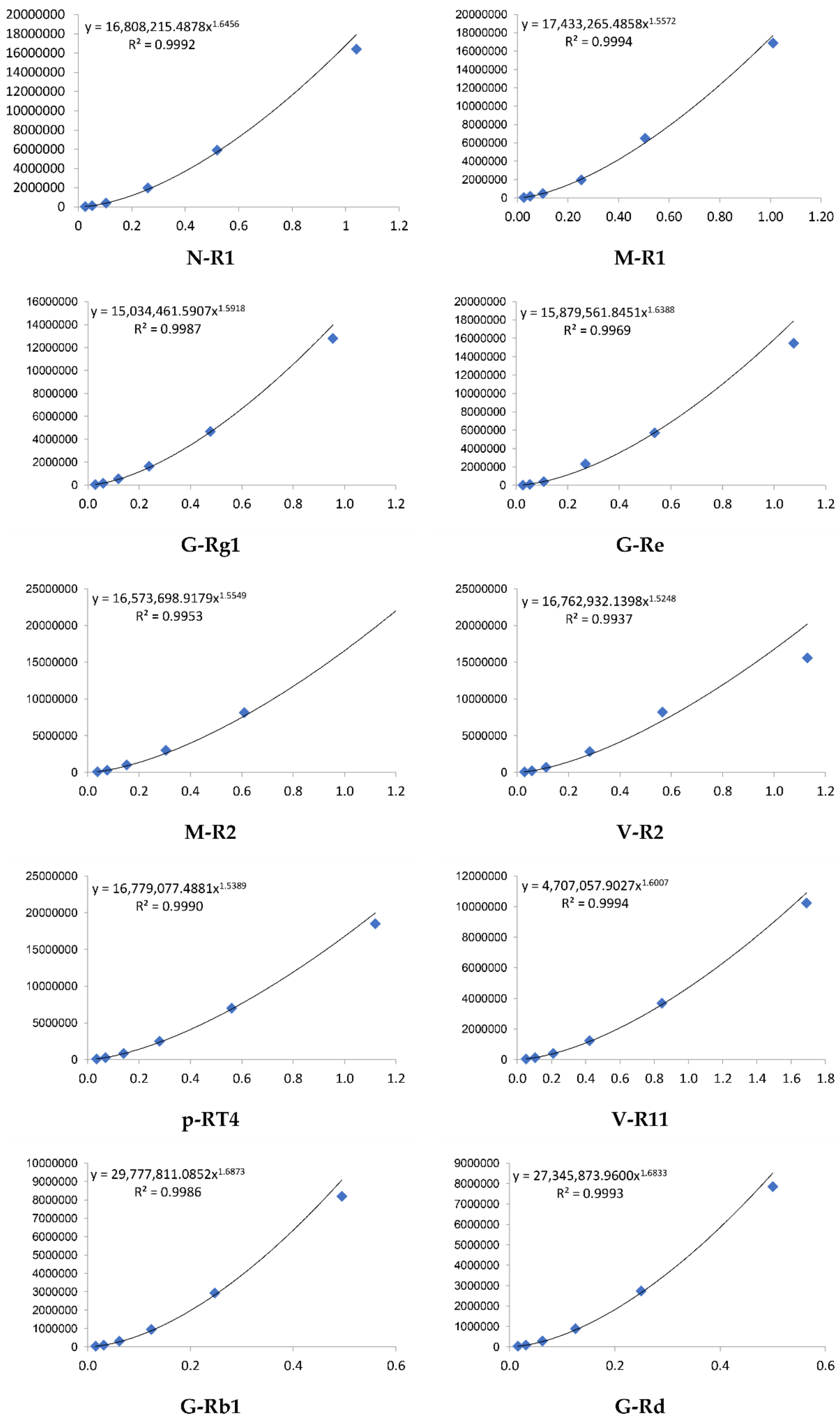

Figure S1. Calibration curve of 10 saponins determined by HPLC-ELSD.

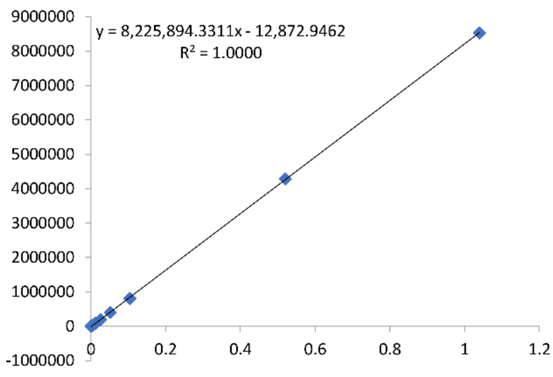

N-R1

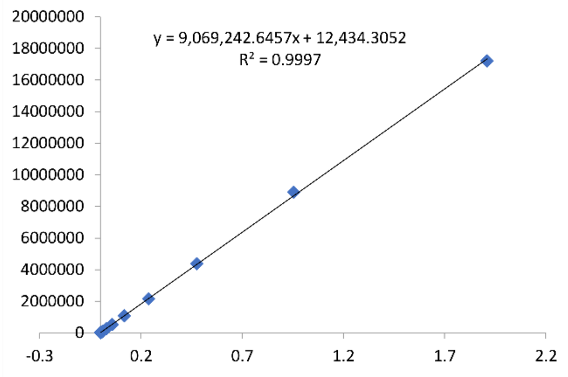

G-Rg1

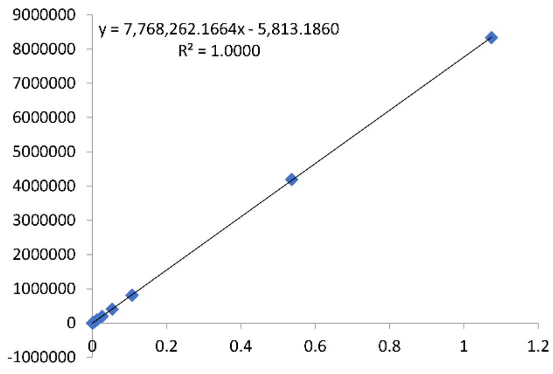

G-Re

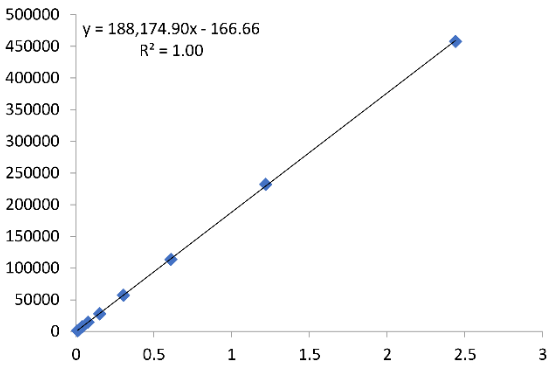

M-R2

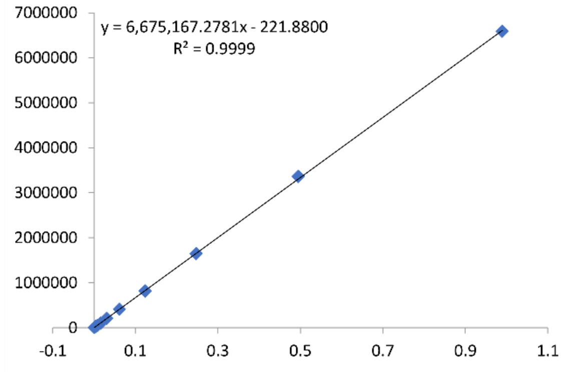

G-Rb1

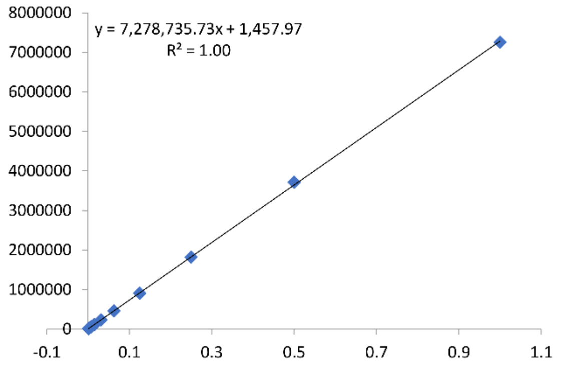

G-Rd

Figure S2. Calibration curve of 10 saponins determined by HPLC-UV.

Table S1. The increase of nebulization gas pressure from 3.5 bar to 3.8 bar could increase the reproducibility of the peak area presented by the decrease of %R.S.D value.

		G-Rg1	M-R2	G-Rb1	G-Rd
3.5 bar	%R.S.D	9.55	6.88	5.81	6.16
3.8 bar	%R.S.D	2.02	1.71	1.76	2.00

Table S2. Saponin contents of *Panax vietnamensis* radix from 2-7 years old detected by HPLC-ELSD. Results are present as mean±SD (n=5).

Age	Saponin content (%, w/w)							
	Rg1	MR2	VR11	p-Rt4	VR2	Rb1	Rd	Total
2	1.38±0.06	1.57±0.37	0.19±0.04	0.23±0.12	0.07±0.01	0.5±0.06	0.27±0.1	4.21±0.56
3	1.92±0.61	1.82±0.51	0.18±0.11	0.22±0.14	0.08±0.01	0.59±0.21	0.47±0.19	5.28±1.68
4	4.63±1.51	5.08±1.28	0.45±0.18	0.81±0.56	0.14±0.06	1.48±0.65	1.35±0.77	13.94±4.52
5	4.61±2.22	5.68±0.92	0.5±0.08	0.94±0.73	0.13±0.02	1.58±0.63	1.37±1.39	12.36±7.08
6	4.17±2.05	4.22±1.57	0.37±0.17	0.45±0.14	0.1±0.03	1.43±0.81	1.51±1.44	12.24±5.78
7	4.7±1.2	6.56±1.77	0.58±0.2	0.73±0.46	0.14±0.04	1.87±0.66	1.65±0.76	16.24±3.54

Table S3. Saponin contents of *Panax vietnamensis* rhizome from 2-7 years old detected by HPLC-ELSD. Results are present as mean±SD (n=5).

**Age**	**Saponin content (%, w/w)**							
	**Rg1**	**MR2**	**VR11**	**p-Rt4**	**VR2**	**Rb1**	**Rd**	**Total**
2	1.4±0.67	1.29±0.59	0.23±0.05	0.27±0.11	0.17±0.02	0.67±0.18	0.99±0.45	5.01±1.82
3	2.09±0.35	2.27±0.57	0.34±0.06	0.46±0.14	0.17±0.01	0.95±0.16	1.09±0.23	7.36±0.95
4	3.67±0.97	3.7±0.74	0.44±0.12	0.56±0.17	0.15±0.03	1.66±0.55	2.9±0.58	13.08±1.89
5	3.69±1.25	4.54±0.88	0.47±0.14	0.88±0.59	0.11±0.03	1.64±0.55	2.35±1.32	13.69±3.35
6	4.24±0.76	4.93±1.53	0.51±0.17	0.77±0.31	0.15±0.04	2.02±0.57	3.48±1.69	16.1±3.73
7	3.83±0.23	5.73±1.93	0.6±0.18	0.55±0.5	0.15±0.04	2.52±0.84	2.67±0.44	16.06±2.72
